# Better movers, better friends? A test for the environmental stress hypothesis in typically developing primary school children

**DOI:** 10.1111/bjdp.70016

**Published:** 2025-09-08

**Authors:** Anne G. M. de Bruijn, Johanna E. A. Brocken

**Affiliations:** ^1^ Faculty of Behavioural and Movement Sciences, LEARN! Research Institute Vrije Universiteit Amsterdam Amsterdam the Netherlands

**Keywords:** developmental theory, emotional development, peer behaviour, perceptual/motor development, social development

## Abstract

Relations between children's motor skills and internalizing problems are poorly understood. The environmental stress hypothesis (ESH), originally developed for motor‐impaired children, may provide understanding, yet has been scarcely examined in typically developing children. Therefore, we examined: (1) relations between children's motor skills and internalizing problems; (2) the role of secondary stressors, specifically interpersonal conflicts and externalizing problems; and (3) the role of personal resources, namely, prosocial behaviour and social self‐efficacy (SSE). About 1154 Dutch primary school children (mean age 9.0 years, 50.0% boys) participated. Multilevel structural equation models showed that children's motor skills were related to internalizing problems, with a weak indirect relation via interpersonal conflicts. SSE had a weak indirect relation with internalizing problems. Prosocial skills (personal resource) and externalizing problems (secondary stressor) did not mediate relations between motor skills and internalizing problems. The ESH seemed applicable in typically developing children, although relations were weaker than for motor‐impaired children.

**Statement of Contribution**

- Children's motor skills are related to internalizing problems, yet relations are poorly understood.
- Peer conflicts and social self‐efficacy explain links between motor skills and internalizing problems.
- The Environmental Stress Hypothesis seems also applicable to typically developing children.

## INTRODUCTION

In recent years, negative trends in children's motor competence have been reported (Mombarg et al., 2023; Schlag et al., 2021; Wessely et al., 2022). These results are worrying, given the importance of well‐developed motor skills for many aspects of physical health (e.g., aerobic fitness, muscular endurance and strength, BMI), physical well‐being, and the building of a lifelong physically active lifestyle (Barnett et al., 2016, 2024; Lloyd et al., 2014; Logan et al., 2015). High levels of motor competence are not only critical for children's physical development; they also predict social–emotional development and functioning (Opstoel et al., 2020; Rodriguez‐Ayllon et al., 2019).

### Fundamental motor skills

Motor competence refers to the degree of proficient performance in various motor skills (Robinson et al., 2015). In early childhood, fundamental motor skills (FMS) are considered important precursors of general motor competence (Clark & Metcalf, 2002; Stodden et al., 2008; Utesch & Bardid, 2019), referring to basic movement patterns such as running or catching (Gallahue & Ozmun, 2006). The early years present a critical period in developing FMS, building a foundation for more specialized and sport‐specific skills (Gallahue & Ozmun, 2006). Moreover, emerging motor skills help children to explore and interact with their environment in increasingly complex ways, enhancing opportunities for cognitive and social–emotional development (Piaget & Inhelder, 1969). By interacting with their environment, children learn to regulate their emotions, such as experiencing fear and dealing with frustration. Also, better‐developed motor skills provide children with opportunities for social interactions with peers (Bar‐Haim & Bart, 2006), being a solid basis for their general social–emotional development (LaFreniere, 2000) by developing prosocial behaviours and building social relationships (Pellegrini & Smith, 1998).

### Social–emotional development

The domain of social–emotional development represents a broad range of behaviours and skills related to the self and relationships with others, amongst others self‐concept, peer relationships, and emotion regulation (Malti & Noam, 2016). Social–emotional development during childhood lays an essential foundation for lifelong development, predicting success across a wide range of life outcomes. Children with a more positive social–emotional development have a higher chance of, amongst others, a successful educational career and employment, good physical and mental health, and reduced risks of substance use and criminal activity (Jones et al., 2015; Moffitt et al., 2011).

Importantly, impairments in motor development are related to developmental problems in the social–emotional domain as well (Cheung et al., 2022; Leonard & Hill, 2014). Developmental problems refer to developmentally inappropriate behaviours that restrict a child in adequate functioning within social contexts, including internalizing problems (e.g., anxiety, social withdrawal) and externalizing problems (e.g., aggression, disruptiveness; Campbell, 1998). Behavioural problems can occur alongside social–emotionally competent behaviours, and the absence of behavioural problems does not necessarily equal social competence (Halle & Darling‐Churchill, 2016), underlining the importance of taking both into account as separate developmental outcomes. Particularly, internalizing problems have been the focus of research (Cairney et al., 2010, 2013; Piek et al., 2010), as these problems typically persist into adulthood (Lingam et al., 2012; Piek et al., 2010), strongly and negatively affecting life quality and functioning (Cairney et al., 2013). Internalizing problems refer to symptoms of depression and anxiety, which can be present independent of diagnosed disorders (Cairney et al., 2010; Piek et al., 2010). In primary school, internalizing problems typically manifest in symptoms such as somatic complaints, separation anxiety, social withdrawal, or low self‐esteem (e.g., Chorpita et al., 2000; Goodman, 1997).

### The elaborated environmental stress hypothesis

The elaborated environmental stress hypothesis (ESH) aims to explain pathways between motor and internalizing problems, specifically focusing on children with developmental coordination disorder (DCD; Cairney et al., 2013), a movement skill disorder characterized by impaired motor coordination (American Psychiatric Association, 2022). DCD strongly affects daily functioning and puts children at risk for psychosocial maladaptation, emotional symptoms, and peer problems (Katagiri et al., 2021). According to ESH, internalizing problems in children with motor difficulties arise primarily from environmental rather than biological factors (Cairney et al., 2010, 2013). The ESH model claims that motor difficulties are primary stressors for children with DCD, resulting in a range of secondary stressors: negative physical and psychological outcomes that substantiate internalizing problems. Cairney et al. (2013) refined this model, including various personal resource mediators that mitigate the effects of secondary stressors, including social and personal resources, such as social networks and prosocial skills.

In line with the thoughts of ESH, children with motor difficulties more often receive negative reactions from their peers, being bullied and stigmatized because of their visible dysfunction (Cairney et al., 2013; Campbell et al., 2012; Losse et al., 1991). Relatedly, they tend to be more isolated, have fewer playmates, and spend more time alone or watching their peers play (Livesey et al., 2011; Schoemaker & Kalverboer, 1994; Smyth & Anderson, 2000). These interpersonal conflicts are enormous psychological stressors evoking strong emotional responses and contributing to internalizing problems (Cairney et al., 2013; Erskine et al., 2024; Gasser‐Haas et al., 2020; Pfeifer & Allen, 2021). Accordingly, interpersonal conflicts have been found to fully mediate the association between motor performance and internalizing problems of children with DCD (Gasser‐Haas et al., 2020; Mancini et al., 2018a; 2018b; Wagner et al., 2012, Wagner et al., 2016).

Where the original ESH model mainly focused on interpersonal conflicts as secondary stressors, more recent studies underline the importance of other interpersonal factors. Specifically, externalizing problems are often mentioned, being defined as antisocial and conduct‐related behaviours, such as aggression and impulsive and oppositional behaviours (Erskine et al., 2024). Children with poor motor skills are more likely to exhibit externalizing problems (Wagner et al., 2016; James et al., 2022), increasing the risk of interpersonal conflicts (McHale et al., 2003). Although limited in scope, there are suggestions that externalizing problems are a mediator in the relation between poor motor skills and internalizing problems as well (de Medeiros et al., 2022).

### Social self‐efficacy and competence as personal resources

Children's personal resources play an important role in mediating negative relations between secondary stressors and internalizing problems. Specifically, social self‐efficacy (SSE) and competence are often mentioned as important mediators in this relation (Erskine et al., 2024). The importance of SSE in children's social–emotional development has been emphasized by social cognitivism, referring to the confidence that children have in their abilities to execute social behaviours (Bandura, 1977b). SSE strongly determines (the results of) children's social behaviours, interactions, and relationships (Asher & Coie, 1990; Kim & Cicchetti, 2010). Accordingly, low feelings of SSE are linked to antisocial behaviour, academic problems, criminality, and school drop‐out (Akelaitis & Lisinskiene, 2018).

Social competence is a related but distinct factor, referring to children's competence in adjusting their behaviour to meet demands of various social contexts (Fabes et al., 2006). Specifically, prosocial behaviours have been identified as an important pathway in the ESH model (Erskine et al., 2024), being defined as the engagement in voluntary and intentional acts that benefit another person, amongst others cooperating, helping, and sharing (Eisenberg & Fabes, 2006). Prosocial behaviours are prerequisites for positive social interactions, social acceptance, and building social relationships, and thus a healthy social adjustment in general (Eisenberg & Fabes, 1990; Gandotra et al., 2023). In accordance with ESH, prosocial behaviours have been identified as a mediator in the relation between poor motor skills and internalizing problems (Wilson et al., 2022). As poor motor skills impede children's engagement in play activities (Holloway & Long, 2019), this limits them in developing prosocial skills, enforcing their existing social problems and exacerbating internalizing problems (Mancini et al., 2016; Poulsen & Ziviani, 2004).

### Relations in typically developing children

Although the ESH model was originally developed to explain experiences of children with DCD, findings are thought to be generalizable towards children with poor motor skills in general (Erskine et al., 2024). Poor motor‐skilled children generally perceive themselves to be less socially competent (Piek et al., 2008; Rigoli et al., 2012), in turn being an important risk factor for internalizing problems (Mancini et al., 2016). Also, it has been suggested that the ESH applies across the motor skill spectrum, meaning that similar relationships apply for children without motor skill problems (Erskine et al., 2024). To date, studies have examined relations between motor skills and social–emotional outcomes in typically developing children, but have not yet explicitly tested the ESH model in this population (Mancini et al., 2016). This is an important area for further research, as the relation between these constructs may be overestimated when focusing on the extreme end of the motor skill spectrum (Rigoli et al., 2012; Salaj & Masnjak, 2022). Yet, there are arguments for why the hypothesized relations of the ESH model can be found in the typically developing population as well.

First, a comparison of relations between motor skills, self‐efficacy, and social–emotional outcomes for children with DCD compared with typically developing children showed that, in both groups, internalizing problems increased as motor skills decreased, making motor skills a crucial determinant of social–emotional development regardless of disability (Lee et al., 2020). Although this study included only a small sample of participants, results are in line with the assumptions of the ESH model.

Second, multiple studies on relations between motor and social–emotional skills have been conducted with typically developing children in the preschool setting. In general, such studies report positive relations between motor skills and social–emotional development (Bar‐Haim & Bart, 2006; Gandotra et al., 2023; Hirata et al., 2021; Piek et al., 2008; Salaj & Masnjak, 2022). Preschoolers with better‐developed motor skills have higher levels of social skills, predicting fewer internalizing problems (Wilson et al., 2013); and the motor skills of young children are related to their social–emotional adjustment (Bart et al., 2007). Similarly, in adolescents, the relation between motor skills and internalizing problems was found to be mediated by self‐concept (Rigoli et al., 2012).

Thirdly, motor skills of typically developing children have been linked to various social–emotional constructs (e.g., social status, social acceptance, bullying, rejection), within regular school contexts (Livesey et al., 2011; Ommundsen et al., 2010), play settings (Livesey et al., 2011) and in physical education (de Bruijn & van der Wilt, 2023; Grimminger, 2013, 2014; Jiménez‐Barbero et al., 2020; Wei & Graber, 2023). These concepts are all strongly linked to children's social–emotional competence and the absence of behavioural problems (Gifford‐Smith & Brownell, 2003; Newcomb et al., 1993).

Although these results point towards the applicability of the hypotheses of the ESH model in typically developing primary school children, the ESH model has not yet been explicitly tested in this population. Such an examination seems vital, as children start to rely less on their family members when entering primary school, making positive social interactions and behaviour of vital importance for children building a social network of their own – with peers and teachers. Also, as children enter primary school, they have a growing ability to accurately assess their own and others' motor competence. For children with poor motor skills, this greatly increases the chance of being excluded from social situations because of their poor motor skills. This may ultimately result in internalizing problems (Øksendal et al., 2022).

### The present study

Although the ESH is considered applicable to typically developing children, research examining this assumption is scarce. Hence, the aim of this study was to examine the interrelatedness of motor and social–emotional skills in primary school children without developmental problems. The ESH includes many interrelated factors, making it extremely complex to test the full model in one single study (Mancini et al., 2016, 2019). For feasibility, we therefore decided to test a subsection of the framework via confirmatory analyses. We excluded the constructs physical inactivity and obesity, which, according to the ESH model, mediate the relation between motor skills and internalizing problems, and moderate the intermediary pathways via personal resources and interpersonal stress (Cairney et al., 2013; Mancini et al., 2016, 2019). Previous findings on the importance of these constructs have been mixed (Erskine et al., 2024), and the psychological distress accompanying poor motor skills is thought to be better accounted for by differences in psychosocial well‐being (Li et al., 2019). To avoid overcomplicating the model, we therefore excluded physical inactivity and obesity. In addition, we did not include the mediating role of social resources (i.e., social support from parents or peers). There is limited evidence for the role of social support in mediating the relation between poor motor skills and internalizing problems in children, probably because the relative importance of social support changes from childhood to adolescence (Cairney et al., 2013; Skinner & Piek, 2001).

In this study, we examine whether children's motor skills are related to their internalizing problems, and whether this relation is mediated by interpersonal conflicts and externalizing problems. In addition, we examine the mediating role of SSE and prosocial skills. First, we hypothesize that more advanced motor skills will be related to lower levels of internalizing problems. Second, we expect this relationship to be significantly mediated by (a) interpersonal conflicts and (b) externalizing problems. Third, we hypothesize that SSE and prosocial skills will mediate both the direct association between motor skills and internalizing problems, as well as the indirect pathways through interpersonal conflicts and externalizing problems.

## MATERIALS AND METHODS

### Design

This study is part of a larger randomized controlled trial testing the effectiveness of a schoolyard intervention on children's physical activity, motor, social–emotional, and cognitive skills. For this cross‐sectional study, only pretest data were used. Data were collected in school year 2023–2024, of which the pretest was conducted at the start of the school year (September–October).

### Participants

Schools were recruited via convenience sampling, using social media and information letters. No specific inclusion criteria were used, but special education schools were excluded from participating. In the end, 21 regular primary schools signed up, with in total, *n* = 1154 Dutch children participating (mean age 9.0 years, SD = 1.3; 577 boys – 50.0%). Schools were located throughout the country, in both rural and urban areas. Children were in grade 2 (*n* = 291, 25.2%), 3 (*n* = 279, 24.2%), 4 (*n* = 242, 21.0%) or 5 (*n* = 342, 29.6%). For all participating children, informed consent was provided by their parents. The study was ethically approved by the ethics committee of the Faculty of Behavioural and Human Movement Sciences of the Vrije Universiteit Amsterdam (VCWE‐2021‐160).

### Instruments

#### Fundamental motor skills

Children's FMS were measured using the Athletic Skill Track (AST; Hoeboer et al., 2016, 2018). The AST presents a track consisting of a series of five to seven concatenated FMS (e.g., balancing, hopping), providing an overall assessment of children's motor competence. Two different tracks were used, depending on children's age (AST‐II for ages 6–9; AST‐III for ages 9–12). Children completed the track as fast as possible, with the time to complete the AST being used as an outcome measure. In cases where a child executed one of the exercises incorrectly, they were required to restart the track, with the timer continuing to run. This procedure led to an increased completion time, thereby yielding a lower motor skill score– this way presenting a measure of both accuracy and speed in executing motor skills. The AST‐II and AST‐III present reliable (test–retest reliability; AST‐II: ICC = 0.802; AST‐III: ICC = 0.800; Hoeboer et al., 2018) and valid (Brocken et al., 2023) measures of children's motor competence. Although typically scores are converted to motor skill quotients based on norm‐referenced values taking into account gender and age, we used raw scores to enhance power. Gender and age differences were taken into account by adding gender and age to the model as covariates.

#### Internalizing and externalizing problems, interpersonal conflicts, and prosocial behaviour

Children's internalizing problems, externalizing problems, interpersonal conflicts, and prosocial behaviour were measured with the Strengths and Difficulties Questionnaire (SDQ; Goodman, 2001) – a short behavioural questionnaire to measure behaviour, emotions, and relationships of children between 2 and 17 years. The SDQ consists of a teacher‐, parent‐, and self‐report form, of which only the teacher report was used for feasibility reasons. In total, the SDQ includes 25 items over 5 scales (5 items each): hyperactivity, emotional symptoms, conduct problems, peer problems, and prosocial behaviour. Items are answered on a 3‐point scale (not true, somewhat true, certainly true), and scores for each subscale are summed. Here, we used scores on the subscales emotional problems (internalizing problems), peer problems (interpersonal conflicts), and prosocial behaviour, ranging from 0 to 10. Following SDQ's protocol, scores on the hyperactivity and conduct problems subscales were summed to get a score for externalizing problems, ranging from 0 to 20. Higher scores indicate more internalizing and externalizing problems and interpersonal conflicts, and higher levels of prosocial behaviour. The SDQ–teacher form is a valid and reliable measure of prosocial behaviour (internal consistency *α* = 0.84, test–retest reliability *α* = 0.74), and social–emotional difficulties (internal consistency *α* = 0.87, test–retest reliability *α* = 0.80; Goodman, 2001).

#### Social self‐efficacy

The Self‐Efficacy Questionnaire for Children (SEQ‐C) was used to measure SSE (Muris, 2001). The SEQ consists of 21 items on three subscales (academic, social, emotional) of which only the SSE subscale was used, comprising seven items scored on a 5‐point Likert scale ranging from not at all (1) to very well (5). An example item is ‘I can become friends with other students of my age’. The SEQ‐C is a valid and reliable measure of children's general self‐efficacy (test–retest *α* = 0.88), providing reliable (*α* = 0.85) and valid scores on the SSE‐subscale specifically (Muris, 2001, 2002).

### Procedure

All tests were administered by research assistants who were trained during a session of 4 h. Motor skills were tested during one PE lesson. Research assistants showed how to perform the track, after which children got three practice attempts. The fourth attempt was timed to be used as an outcome score. The SEQ‐C was administered on the same day, in the children's classroom. Research assistants provided instructions, after which children individually completed the questionnaire. Classroom teachers filled out the SDQ for all participating children in their class within the same period.

### Analyses

Data was preprocessed in IBM SPSS Statistics 29.0, showing that data were not missing completely at random (Little MCAR test: *χ* ^2^(17) = 59.50, *p* < .001). Data were complete for 1015 of the cases (88.0%); with MQ‐scores missing for 50 participants (4.3%), SEQ‐C scores missing for 57 cases (4.9%); both MQ‐scores and SEQ‐C scores missing for 20 cases (1.7%); SDQ‐data missing for 10 cases (0.9%), and data on all outcomes missing for two cases (0.2%). Full‐information Maximum Likelihood (FIML) Estimation in Mplus (Muthén & Muthén, 2017) was used to account for missing data, using all available data to compute a likelihood function for each participant. FIML is widely considered an appropriate and effective method for handling missing data, as it yields efficient, robust, and unbiased parameter estimates, particularly when data are MCAR or missing at random (MAR; Enders, 2010). However, results from Little's MCAR test indicate that the assumption of MCAR does not hold in our data, leaving the precise mechanism of missingness uncertain. As it is not statistically possible to distinguish between MAR and missing not at random (MNAR; Heymans & Twisk, 2022), the true nature of the missingness remains unknown. Nevertheless, even under such uncertainty, FIML remains the recommended approach, as it has been shown to produce acceptably biased estimates when the proportion of missing data is relatively low (Tang & Tong, 2024).

Next, we tested the suggested factor structure in Mplus. A single‐indicator factor was created for FMS to account for measurement error of a unidimensional measure. FMS was indicated by the AST score, with the corresponding factor loading fixed at 1 and indicator error variance fixed at the product of the measure's sample variance (VAR(X)) and 1–*ρ*, where *ρ* refers to the reliability of the measure (*α* = 0.80 for the AST; Hoeboer et al., 2018). Latent factors were constructed for interpersonal conflicts and externalizing problems (secondary stressors), SSE and prosocial behaviour (personal resource mediators), and internalizing problems, made up of their corresponding items.

This factor structure was used to test three multilevel structural equation models. The first model tested only direct relations of motor skills, secondary stressors, and interpersonal resources with internalizing problems. In a second model, indirect relations between motor skills and internalizing problems via secondary stressors (interpersonal conflicts and externalizing problems) were tested (i.e., the mediating role of secondary stressors). Personal resource mediators (SSE and prosocial behaviours) were only related to internalizing problems. In a third model, indirect relations between motor skills, secondary stressors, and internalizing problems via personal resource mediators (SSE and prosocial behaviour) were included as well (i.e., testing whether personal resource mediators mediated the indirect relationships between motor skills and internalizing problems via secondary stressors). In all models, gender and age were added as covariates (Erskine et al., 2024).

Weighted least squares means and variances adjusted (WLSMV) estimation was used to test the models. Model fit was assessed with the root mean square error of approximation (RMSEA), comparative fit index (CFI), and Tucker–Lewis Index (TLI), using cut‐offs of 0.06, 0.90, and 0.90 respectively (Hu & Bentler, 1998). The conventional *χ* ^2^ statistic is reported as well, but was not used for assessing model fit, given that it is strongly affected by sample size and model complexity (Hu & Bentler, 1998). Standardized estimates of path coefficients (Beta‐values) and corresponding p‐values and confidence intervals were used for significance testing.

## RESULTS

Table 1 presents descriptive statistics of included variables. Correlations between included (latent) variables are presented in Table 2.

**TABLE 1:** Descriptive statistics of latent variables.

	Mean (SD)	Min–max
Motor skills	89.58 (16.52)	40–148
Interpersonal conflicts	1.09 (0.17)	0–10
Externalizing problems	3.76 (0.24)	0–15
Internalizing problems	2.51 (0.27)	0–10
Prosocial behaviour	8.14 (0.49)	0–10
Social self‐efficacy	3.69 (0.52)	1–5

**TABLE 2:** Correlations between included (latent) variables.

	1	2	3	4	5	6	7	8
1. Motor skills	–							
2. Interpersonal conflicts	−0.14**	–						
3. Externalizing problems	−0.02	0.71***	–					
4. Internalizing problems	−0.18**	0.44***	0.38***	–				
5. Prosocial behaviour	0.03	−0.74***	−0.86***	−0.30***	–			
6. Social self‐efficacy	0.15*	−0.29***	−0.17***	−0.21***	0.22**	–		
7. Gender	−0.03	−0.06	−0.23***	0.08*	0.26***	0.02	–	
8. Age	−0.18*	0.11	0.01	−0.01	−0.03	0.05	−0.05*	–

*Note*: *p < .05; **p < .01; ***p < .001.

### Direct relations

A model with only direct relations between motor skills, secondary stressors (interpersonal conflicts, externalizing problems), personal resource mediators (SSE, prosocial behaviour), and internalizing problems did not provide a good fit when comparing model fit indices to the set thresholds (χ^2^(563) = 2093.72, *p* < .001, RMSEA = 0.05, CFI = 0.83, TLI = 0.81, SRMR = 0.109). Modification indices suggested adding indirect relations of secondary stressors and personal resource mediators to improve model fit.

### Model with added indirect relations via secondary stressors

A second model with added indirect relations between motor skills and internalizing problems via secondary stressors (interpersonal conflicts, externalizing problems) was examined. Personal resource mediators (SSE, prosocial behaviour) were included in this model, but only direct relations with internalizing problems were included to specifically test the added value of mediating pathways via secondary stressors. Again, this model did not provide an acceptable fit when comparing model fit indices to the set thresholds (χ^2^(563) = 2093.72, *p* < .001, RMSEA = 0.05, CFI = 0.83, TLI = 0.81, SRMR = 0.109). Modification indices suggested adding indirect relations of personal resource mediators to improve model fit.

### Model with added indirect relations via personal resource mediators

**FIGURE 1:**
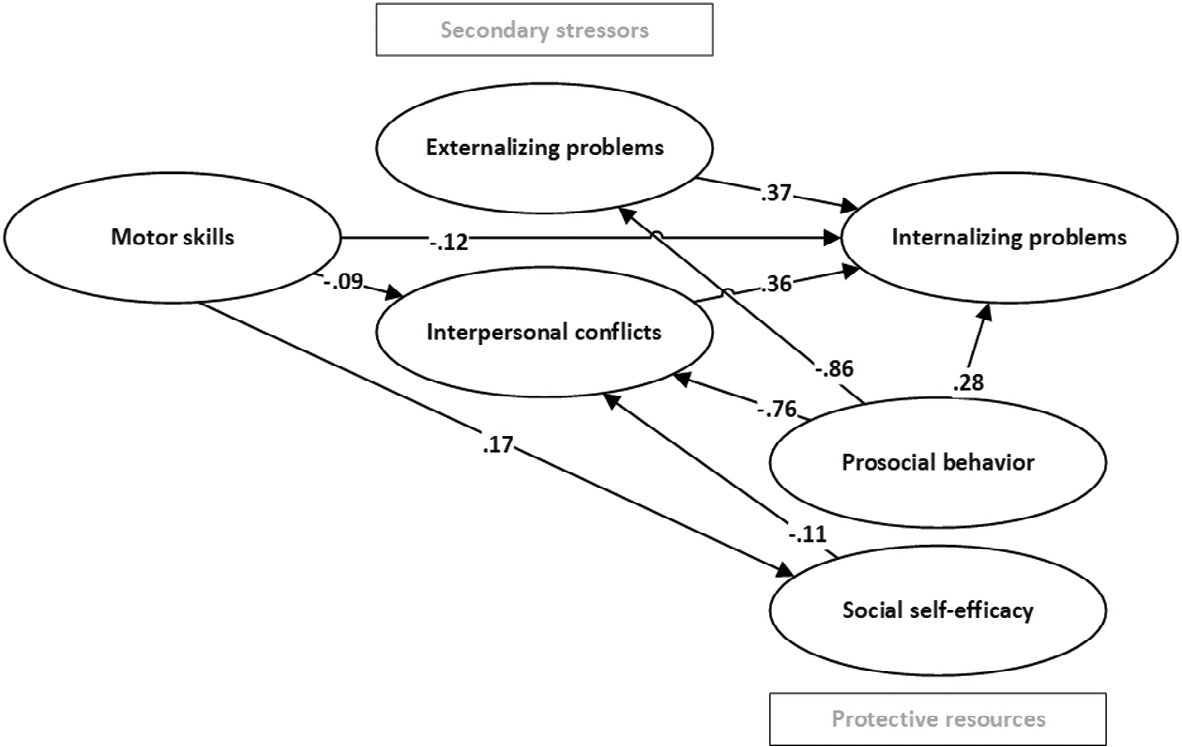
Visual representation of significant paths between independent and dependent variables. Standardized path coefficients (betas) are presented in the figure.

In a third model, relations were added between personal resource mediators (SSE, prosocial behaviour), motor skills, and secondary stressors (interpersonal conflicts, externalizing problems), resulting in an acceptable model fit, comparing model fit indices to the set cut‐off points (χ^2^(555) = 1397.27, *p* < .001, RMSEA = 0.04, CFI = 0.91, TLI = 0.90, SRMR = 0.049). In total, the included factors explained 26.2% (SE = 0.04, *p* < .001) of the outcome internalizing problems. Significant relations are presented in Figure 1. The full model including non‐significant relations is presented in Appendix S1.

#### Direct relations

Motor skills were significantly and negatively related to internalizing problems (*β* = −0.12 (0.04), *p* = .004, 95% CI: −0.20 to −0.04). Externalizing problems (*β* = 0.37 (0.14), *p* = .009, 95% CI: 0.09 to 0.64) and interpersonal conflicts (*β* = 0.36 (0.13), *p* = .006, 95% CI: 0.10 to 0.62) were both positively related to internalizing problems. Unexpectedly, prosocial behaviour was positively related to internalizing problems (*β* = 0.28 (0.11), *p* = .010, 95% CI: 0.07 to 0.49), whereas the relation between SSE and internalizing problems was not significant (*β* = −0.08 (0.04), *p* = .07, 95% CI: −0.16 to 0.01). Children with poorer motor skills, more externalizing problems and interpersonal conflicts, and more prosocial behaviours experienced more internalizing problems.

Prosocial behaviour was significantly and negatively related to externalizing problems (*β* = −0.86 (0.03), *p* < .001, 95% CI: −0.91 to −0.81): children with fewer prosocial behaviours showed more externalizing problems. There were no significant relations between externalizing problems and either motor skills (*β* = −0.004 (0.04), *p* = .91, 95% CI: −0.08 to 0.07) or SSE (*β* = 0.02 (0.04), *p* = .53, 95% CI: −0.05 to 0.09). In total, a significant 73.4% of externalizing problems was explained (SE = 0.05, *p* < .001).

Interpersonal conflicts were significantly and negatively related to motor skills (*β* = −0.09 (0.03), *p* = .01, 95% CI: −0.15 to −0.03), SSE (*β* = −0.11 (0.05), *p* = 0.03, 95% CI: 0.22 to −0.01), and prosocial behaviour (*β* = −0.76 (0.04), *p* < .001, 95% CI: −0.84 to −0.67). Children with better motor skills, higher SSE, and more prosocial behaviour had fewer interpersonal conflicts. A significant amount of 61.1% of variance of interpersonal conflicts was explained (SE = 0.04, *p* < .001).

Motor skills were significantly and positively related to SSE (*β* = 0.17 (0.06), *p* = .01, 95% CI: 0.05 to 0.29): Children with better‐developed motor skills had higher levels of SSE. A non‐significant amount of variance of SSE was explained (*R* ^2^ = 0.03, SE = 0.02, *p* = .15). Motor skills were not significantly related to prosocial behaviour (*β* = 0.04 (0.05), *p* = .48, 95% CI: −0.07 to 0.14), although a significant amount of 6.9% of the variance in prosocial behaviour was explained (SE = 0.02, *p* < .001).

#### Indirect relations via secondary stressors

As expected, motor skills were indirectly and negatively related to internalizing problems via interpersonal conflicts, representing a weak relation (*β* = −0.03 (0.02), *p* = .040, 95% CI: −0.06 to −0.01): children with better‐developed motor skills experienced fewer interpersonal conflicts and lower levels of internalizing problems. Unexpectedly, the indirect relation between motor skills and internalizing problems via externalizing problems was not significant (*β* = −0.002 (0.01), *p* = .91, 95% CI: −0.03 to 0.03).

#### Indirect relations via personal resource mediators

##### Social self‐efficacy

The relation between SSE and internalizing problems was fully mediated by interpersonal conflicts, representing a weak relation (*β* = −0.04 (0.02), *p* = .04, 95% CI: −0.08 to −0.01): children with higher levels of SSE experienced fewer interpersonal conflicts, relating to lower levels of internalizing problems.

Although the indirect relation between motor skills and internalizing problems via SSE was not significant (*β* = −0.01 (0.01), *p* = .12, 95% CI: −0.03 to 0.004), a multiple mediation was found, which followed our expectations. SSE significantly mediated the indirect relation between motor skills and internalizing problems via interpersonal conflicts, representing a weak relation (*β* = −0.01 (0.01), *p* = .04, 95% CI: −0.01 to 0.00): children with better motor skills had higher levels of SSE, relating to fewer interpersonal conflicts and lower levels of internalizing problems.

As self‐efficacy was not significantly related to externalizing problems, SSE was not a significant mediator in the indirect relation between motor skills and internalizing problems via externalizing problems (*β* = 0.001 (0.002), *p* = .47, 95% CI: −0.002 to 0.01), which was against our hypothesis.

##### Prosocial behaviour

Results only provided evidence for a part of the expected mediating role of prosocial behaviour, as prosocial behaviour was indirectly related to internalizing problems via interpersonal conflicts (*β* = −0.27 (0.11), *p* = .011, 95% CI: −0.48 to −0.06) and externalizing problems (*β* = −0.32 (0.12), *p* = .011, 95% CI: −0.56 to −0.07). Children with more prosocial behaviours had fewer interpersonal conflicts and externalizing problems relating to fewer internalizing problems. Yet, as prosocial behaviour was not significantly related to motor skills, prosocial behaviours did not play a mediating role in the relation between motor skills and internalizing problems via interpersonal conflicts (*β* = −0.01 (0.01), *p* = .46, 95% CI: −0.04 to 0.02) or externalizing problems (*β* = −0.01 (0.02), *p* = .54, 95% CI: −0.05 to 0.03), which was against our hypothesis.

## DISCUSSION

We aimed to extend findings of previous studies on the ESH model in children with motor skill problems to the population of typically developing children by examining the relation between children's motor skills and their internalizing problems, and the mediating role of the secondary stressors interpersonal conflicts and externalizing problems herein. We hypothesized that better‐developed motor skills would be related to fewer internalizing problems, with this relation being – at least partly – mediated by fewer interpersonal conflicts and externalizing problems. In addition, we tested the mediating role of prosocial behaviour and SSE, expecting that better‐developed motor skills would relate to higher levels of prosocial skills and SSE, which would, in turn, be related to fewer interpersonal conflicts, lower levels of externalizing problems, and fewer internalizing problems.

Our results showed that children's motor skills were both directly and indirectly, via interpersonal conflicts, linked to internalizing problems: children with better‐developed motor skills had fewer internalizing problems, partly because they experienced fewer interpersonal conflicts (although it should be noted that this relation was only weak, yet significant). In addition, we found SSE to be a personal resource mediator (again a weak, yet significant relation), as children with better motor skills had a higher SSE, in this way experiencing fewer interpersonal conflicts, being related to lower levels of internalizing problems. Unexpectedly, children's prosocial behaviours were positively related to their internalizing problems. Also, although children with more prosocial behaviours seemed to have fewer internalizing problems when they experienced fewer interpersonal conflicts and externalizing problems, prosocial behaviours were not significantly related to children's motor skills, and thus did not act as a personal resource mediator in the relation between motor skills and internalizing problems. Externalizing problems did not mediate the relation between motor skills and internalizing problems and thus were not a secondary stressor in typically developing children.

### The role of secondary stressors

In line with our expectations, children with poor motor skills experienced more interpersonal conflicts (i.e., secondary stressors), linking to higher levels of internalizing problems. Motor problems put children at risk for social difficulties, being ridiculed or excluded from activities because they do not possess the skills for successful participation. Social difficulties in turn are major psychological stressors that strongly increase the risk of internalizing problems (Cairney et al., 2013; Erskine et al., 2024; Gasser‐Haas et al., 2020; Pfeifer & Allen, 2021). This mediating relation was only weak, suggesting that it may be less strongly applicable in typically developing children compared with their motor‐impaired peers.

Externalizing problems were not a secondary stressor, as they were not significantly related to motor skills in this population. This contradicts relations assumed in the ESH model and findings in line with this assumption in children with DCD (de Medeiros et al., 2022; Wagner et al., 2016). Possibly, externalizing problems play a different role for typically developing children compared with their peers with DCD. Externalizing problems are quite common in childhood, with a worldwide prevalence rate amongst children of 5.7% (Polanczyk et al., 2015) and an estimated self‐reported 6.5% of children in the Netherlands displaying some level of externalizing problems (Husky et al., 2018). Yet, in the Netherlands, many children with externalizing problems attend special primary schools (Stoutjesdijk et al., 2012; van der Veen et al., 2010; Zweers et al., 2019), and prevalence rates in regular primary schools (i.e., our target population) are substantially lower. Indeed, descriptives in our sample indicated that teachers reported low levels of externalizing problems, with little variation between children. ESH's assumption of externalizing problems being a secondary stressor may thus not necessarily apply in the population of typically developing children, supporting ideas of relations within the ESH being more pronounced in children at the lowest end of the motor skill spectrum (Rigoli et al., 2012; Salaj & Masnjak, 2022). More research directly comparing typically developing and motor‐impaired populations in a unified model is needed to better understand relational differences between populations. Also, externalizing problems are not often included in examinations of the ESH, underlining a need for further research (de Medeiros et al., 2022; Erskine et al., 2024).

### Social self‐efficacy and prosocial behaviour as personal resource mediators

A higher SSE helped children with poor motor skills in experiencing fewer interpersonal conflicts, consequently being related to fewer internalizing problems, thus presenting a possible underlying mechanism for explaining internalizing problems of children with poor motor skills. This result is in line with previous findings of lower levels of self‐perceived competence in children with DCD (Cairney et al., 2013), and in typically developing adolescents (Rigoli et al., 2012; Skinner & Piek, 2001) Our study extends these findings, reporting that similar processes seem to apply in typically developing children, also extending to perceived competence in the social domain. Again, this mediating relation was only weak and may thus hold to a lower extent in typically developing children compared with their motor‐impaired peers.

Children with better‐developed motor skills are likely to feel more efficacious in social situations because they have had more positive social experiences (Skinner & Piek, 2001). Children with poor motor skills, on the other hand, are likely to be bullied and excluded because of their inability to successfully participate in (physical) activities (Campbell et al., 2012), this way increasing their insecurity in engaging in social encounters (Bar‐Haim & Bart, 2006; Erskine et al., 2024; Jarus et al., 2011; Smyth & Anderson, 2000). In addition, poor motor skills make physical activities more cognitively demanding, thus increasing feelings of incompetence (Smyth & Anderson, 2000). This anxiety will consequently also affect these children's social behaviours (e.g., taking less initiative in approaching others, being more by themselves, interpreting others' behaviours as negative or threatening) (Bar‐Haim & Bart, 2006; Jarus et al., 2011), explaining why they are more likely to experience interpersonal conflicts.

Unexpectedly, SSE was only linked to interpersonal conflicts, whereas no relations with externalizing problems were found. Self‐efficacy relates to the way a situation is perceived, and the coping strategies selected to handle the specific situation. Children with low levels of SSE have a lack of confidence in their ability to respond appropriately to social situations, resulting in maladaptive coping styles, such as self‐blame, worry, or aggressive behaviour (Kim & Cicchetti, 2003), which is why we expected SSE to be related to externalizing problems. Yet, previous studies have reported non‐significant relations between socialself‐efficacy and externalizing problems as well (Cherewick et al., 2023; Kim & Cicchetti, 2003), suggesting that SSE may be a more important resource in cases of internalizing compared with externalizing problems. As only a few studies have examined the relation between SSE and externalizing problems, more research is needed to better understand whether and how SSE relates to children's behavioural problems.

Contrary to our expectations, prosocial skills did not have a similar indirect role in the relation between motor skills and internalizing problems. Although prosocial skills were related to interpersonal conflicts and internalizing problems, they were not significantly linked to motor skills. Most research reporting relations between motor and social skills is based on special populations, such as children with DCD (Bar‐Haim & Bart, 2006; Cairney et al., 2010, 2013; Jarus et al., 2011; Piek et al., 2008; Skinner & Piek, 2001), whereas relations seem to be less pronounced in typically developing children (Rigoli et al., 2012; Salaj & Masnjak, 2022). Possibly, these children have alternative ways of engaging in social experiences that are less reliant on appropriate motor skill levels (e.g., fantasy play, drawing or other creative activities, music), relying on other skills such as verbal communication to achieve social engagement, and they may choose to participate solely in activities they find themselves skilled in (Bar‐Haim & Bart, 2006). Further research examining how children's motor and social skills are related to their recess activities is needed to corroborate such ideas.

Moreover, unexpectedly, prosocial behaviour was positively associated with internalizing problems, suggesting that higher levels of prosocial behaviour may be linked to an increased risk of internalizing problems. Prosocial behaviour has often been regarded as an important resource for engaging in positive social interactions, reducing the risk for anxiety and depression (Zondervan‐Zwijnenburg et al., 2022). In line with these ideas, studies generally indicate that prosocial behaviour is related to lower levels of internalizing problems (Memmott‐Elison et al., 2020). Yet, these effects are small, and relations appear to be complex (Flynn et al., 2015; Huber et al., 2019; Memmott‐Elison et al., 2020; Nantel‐Vivier et al., 2009) as prosocial behaviours can co‐occur with trajectories of both low and high internalizing problems (Nantel‐Vivier et al., 2009). In explaining these controversial results, it has been argued that high levels of prosocial behaviour can lead to an overconcern or worry for others, which may cause internalizing problems (Eisenberg et al., 2024; Hay & Pawlby, 2003; Nantel‐Vivier et al., 2009). Children who are overly concerned with others' emotions and opinions may tend to overlook their own needs, contributing to feelings of anxiety and depression (Morneau‐Vaillancourt et al., 2025). For a precise understanding of the relations between prosocial behaviour and internalizing problems, it is essential that studies start examining the mechanisms underlying the joint development of prosocial skills and mental health (Nantel‐Vivier et al., 2009). The exact relations between prosociality and internalizing problems are thought to differ depending on the type of prosocial behaviour involved, the specific internalizing problems being examined, and background characteristics of the individual child (e.g., gender, age; (Eisenberg et al., 2024; Nantel‐Vivier et al., 2009)). Possibly, the strength and direction of these relations differ depending on the population examined, meaning that differential relations may exist for children with DCD compared with their typically developing peers. A direct comparison of the hypothesized relations in the ESH model is needed to better understand the extent to which (parts of) the model may be specifically.

### Strengths and limitations

Strengths of this study include the large sample of typically developing children in regular primary schools. Also, by constructing multilevel structural equation models, we were able to conduct strong quantitative analyses, taking into account measurement error by constructing latent factors, whilst simultaneously controlling for the nested nature of the data. Yet, results should also be interpreted in light of some limitations.

First, we were unable to identify causal relations because of our cross‐sectional data. Although the directionality of the ESH model has been supported by previous research, few experimental or longitudinal studies exist on how relations between the different factors in the model develop over time (Erskine et al., 2024). Future studies could, for example, implement intervention programmes targeting motor development to examine whether improvements in motor skills lead to reductions in interpersonal conflicts and externalizing problems—potentially through enhanced SSE and prosocial skills—and whether these changes, over time, contribute to fewer internalizing problems and improved mental health. There are suggestions that the strength and the pattern of relations between motor skills and social–emotional outcomes change throughout development (Mancini et al., 2019). Children encounter different developmental challenges as they grow up, requiring different skills, meaning that the importance of motor and social–emotional skills may change as well. Further research following children over longer periods of time is needed to better understand the applicability of the ESH model throughout development.

Secondly, we only included personal resources (SSE, prosocial skills) as mediators, whereas social resources have been identified as an important mediator in the negative relations with secondary stressors as well (Erskine et al., 2024). The experience of social support (i.e., healthy relationships with parents and peers) has been found to mediate negative relations between poor motor skills and internalizing problems by reinforcing a more positive perception of stressful circumstances (Rigoli et al., 2017; Wagner et al., 2016), with particularly parental support being an important social resource for children (Mancini et al., 2016; Rueger et al., 2010; Stewart & Suldo, 2011; Taylor et al., 2020). Given that social support may serve as a critical intervention strategy, further research is necessary to investigate the role of personal and social resources concurrently, identifying both psychological and socio‐cultural factors that could mediate the adverse relations with poor motor skills. It has been argued that the relative importance of social support changes from childhood to adolescence, with adolescents demonstrating a greater need for supportive relationships with parents and peers than children (Cairney et al., 2013; Skinner & Piek, 2001). Therefore, longitudinal studies are essential to truly understand how and when social support can mediate negative relations between poor motor skills and internalizing problems. A strong social network provides opportunities for developing social skills that can act as a mediator in these negative relations, this way being linked to children's personal resources as well (Erskine et al., 2024).

Third, we focused on gross motor skills, whereas the profoundness of children's fine motor skills for social–emotional development has been established, with some studies reporting even stronger relations between fine motor skills, such as handwriting, and social–emotional development compared with gross motor skills (Kim et al., 2016; MacDonald et al., 2017; Salaj & Masnjak, 2022). Especially in the school context, adequate fine motor skills are a facilitator of children's cognitive, academic, and social–emotional development (Salaj & Masnjak, 2022). Many activities in which children engage rely on fine motor skills, such as writing, picking up small objects, using utensils, and working with scissors. At school, children often perform these tasks whilst simultaneously interacting with their environment, including peers and teachers. To allocate sufficient attentional capacity to these social demands, fine motor skills need to be automatized to a certain degree, thereby freeing up cognitive resources for successful social interactions. Conversely, children who have not yet automatized their fine motor skills must devote considerable attention to managing motor demands, which reduces the cognitive resources available for the development of their social–emotional skills (Kim et al., 2016). Relations between gross motor skills and social–emotional outcomes may thus attenuate when also taking into account fine motor skill level.

Lastly, although we included a large, representative sample of Dutch primary school students, we relied solely on convenience sampling, meaning that selection bias may have influenced our results. Also, the generalizability of these findings to non‐Western cultures remains uncertain, as this type of research is typically conducted in Western countries, thereby making results not readily applicable in non‐Western cultures. For future studies it would be interesting to see whether similar relations can be found in, for example, more collectivistic cultures such as in Asia.

### Implications

Although the causality of the relations needs to be proven, our results suggest that poor motor‐skilled children's beliefs regarding their social skills are more important determinants of internalizing problems than their *actual* skill levels. To combat internalizing problems in children with poor motor skills, it thus seems especially important to target these social insecurities, by providing them with successful social experiences by reinforcing appropriate social behaviours with positive feedback, by modelling effective social behaviours, and by acknowledging their emotional reactions during social (inter)actions (Bandura, 1977a). These strategies are thought to strengthen children's resilience and enhance children's feelings of competence for engaging in social activities (Bandura, 1977a), which helps them practice their social skills and build positive peer relationships that can reduce internalizing problems (Erskine et al., 2024). Indeed, interventions aimed at improving SSE have been found to be effective, especially when, with the right dosage, teaching children what the concept entails and including cognitive‐emotional components such as exercises aimed at improving communication, problem‐solving, and emotion recognition (de Mooij et al., 2020; Durlak et al., 2010). Notably, intervention programmes are assumed to work best for those children that have not yet adequately developed their SSE, emphasizing the importance of administering programmes to those that are most in need (de Mooij et al., 2020).

In addition, as well‐developed motor skills provide opportunities for participating in social activities and play (Bar‐Haim & Bart, 2006; Smyth & Anderson, 2000), stimulating children's motor skill development seems essential as well, and can have positive effects on children's social–emotional development (Lee et al., 2016). This can be achieved by targeted training, focusing on the development of FMS that serve as the foundation for more advanced sport‐specific skills (Zhang et al., 2024). By developing these foundational skills, children can more easily participate in a variety of activities during recess, providing opportunities for further development of their motor skills, increasing social skills and building positive peer relationships (Bar‐Haim & Bart, 2006; Haapala et al., 2014; Mak & Koustova, 2023; Pellegrini & Smith, 1998; Smyth & Anderson, 2000), all important determinants of good social–emotional health and well‐being (Cheung et al., 2022; LaFreniere, 2000; Leonard & Hill, 2014).

### Conclusion

Results of our study underline the applicability of ESH in typically developing children, although relations seem to be less pronounced than in children with motor difficulties. Typically developing children with poor motor skills were more at risk for internalizing problems, partly because they experienced more interpersonal conflicts. Externalizing problems did not explain this relation. SSE, but not prosocial behaviour, was a personal resource mediator herein, as children with higher SSE experienced fewer interpersonal conflicts and internalizing problems. To protect children with poor motor skills from experiencing internalizing problems, it seems critical to target their perceptions of social efficacy, which can help them in experiencing fewer social conflicts and internalizing problems. In addition, directly targeting children's motor skills may be beneficial, since well‐developed motor skills provide opportunities for successful participation in social activities. Such efforts are essential, given the detrimental effects that internalizing problems have on children's long‐term social–emotional health and well‐being.

## AUTHOR CONTRIBUTIONS

**Anne G. M. de Bruijn:** Conceptualization; investigation; funding acquisition; writing – original draft; methodology; validation; visualization; formal analysis; project administration. **Johanna E. A. Brocken:** Investigation; writing – review and editing; data curation; project administration.

## CONFLICT OF INTEREST STATEMENT

The authors declare no conflicts of interest.

## Supporting information


Appendix S1.

## Data Availability

The data that support the findings of this study are available from the corresponding author upon reasonable request.
