# Supplementary material for: Better movers, better friends? A test for the environmental stress hypothesis in typically developing primary school children
Source: Br J Dev Psychol. 2025 Sep 8;44(1):217–36. doi: 10.1111/bjdp.70016 (PMC12884367; doi:10.1111/bjdp.70016)
Supplement: Supplementary file 1 — Appendix S1. [file BJDP-44-217-s001.docx]

**Appendix 1: Full model**

**Figure 1.** *Full SEM-model including relations among motor skills, secondary stressors externalizing problems and interpersonal conflicts, protective resources prosocial behavior and social self-efficacy, and internalizing problems, controlling for age and gender. Standardized path coefficients (betas) and associated standard errors are presented in the figure. Dashed lines indicate non-significant relations.*

**
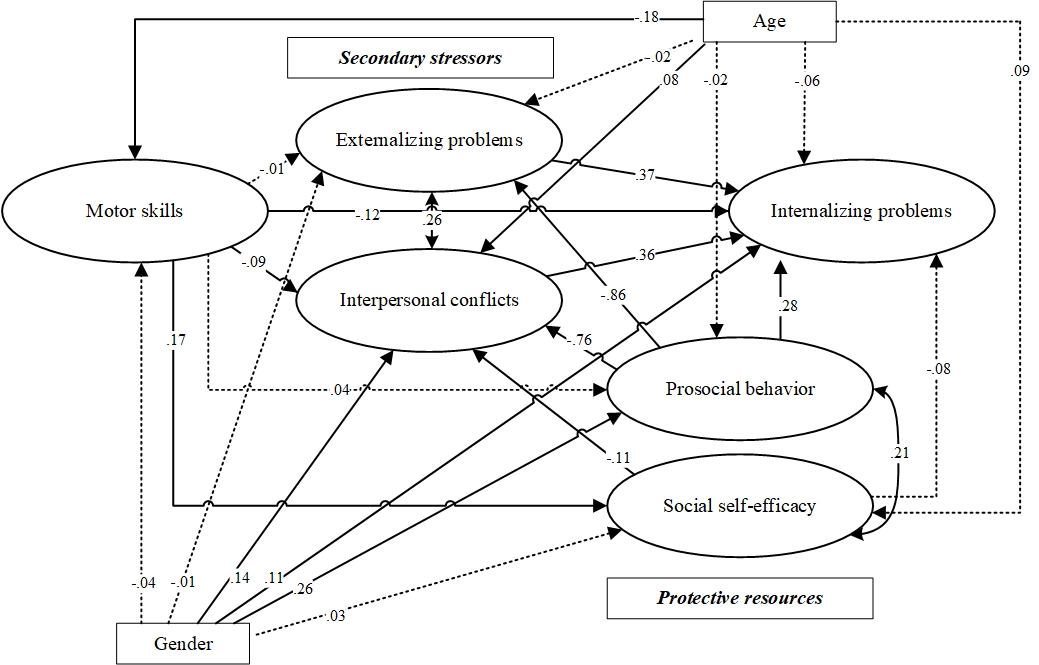
**
